# Implementation fidelity of a multisite maternity waiting homes programme in rural Zambia: application of the conceptual framework for implementation fidelity to a complex, hybrid-design study

**DOI:** 10.1136/bmjph-2024-001215

**Published:** 2025-01-16

**Authors:** Thandiwe Ngoma, Jeanette L Kaiser, Allison J Morgan, Taryn Vian, Davidson H Hamer, Peter C Rockers, Viviane Sakanga, Godfrey Biemba, Misheck Bwalya, Nancy A Scott

**Affiliations:** 1 Deparment of Research, Right to Care Zambia, Lusaka, Zambia; 2 Department of Global Health, Boston University School of Public Health, Boston, Massachusetts, USA; 3 School of Nursing and Health Professions, University of San Francisco, San Francisco, California, USA; 4 School of Infectious Diseases, Department of Medicine, Boston University School Of Medicine, Boston, Massachusetts, USA; 5 AMREF Health Africa, Lusaka, Zambia; 6 National Health Research Authority, Pediatric Centre of Excellence, Lusaka, Zambia; 7 Mothers2mothers Zambia, Lusaka, Zambia

**Keywords:** Public Health, methods, Preventive Medicine

## Abstract

**Background:**

Implementation fidelity measures are critical for understanding complex interventions. Though maternity waiting homes (MWHs) are recommended by the WHO and have been used to help pregnant women access health facilities for decades, a gap exists regarding fidelity studies on MWHs. To better understand intervention outcomes results, we assessed the fidelity of implementation of an improved Core MWH Model in 10 facilities in rural Zambia.

**Methods:**

We analysed indicators for fidelity employing a widely used conceptual framework. We compared performance from October 2016 to July 2018 to goals set out during intervention design. The Core MWH Model consists of three pillars—infrastructure, policies and linkages to care—each designed to be culturally appropriate and responsive to community standards for safety and comfort.

**Results:**

70% of sites exhibited high adherence to the Core MWH Model components. User experience corroborated poorer performing elements: insufficient lighting, small cooking spaces, non-locking cabinets and few educational classes. Mission statements and governing documents were not always visible or available. The proportion of 3206 users who came from>10 km away was higher than the proportion of the surrounding population living at that distance except in two sites with low populations of remote-living women. Women stayed for just below the target of 14 nights. MWH occupancy rate overall was lower (52%) than the target (80%). MWH users reported high quality and satisfaction. Only three MWHs reached 50% female membership on their governance committees but met other key indicators for community ownership and engagement.

**Conclusions:**

This fidelity evaluation of an MWH model in rural Zambia helps bridge the evidence–practice gap for the WHO’s recommendation on implementing MWHs and adds to the expanding body of literature on implementation fidelity studies in global health.

**Trial registration number:**

NCT02620436.

**WHAT IS ALREADY KNOWN ON THIS TOPIC**

- Maternity waiting homes (MWHs) are a WHO-recommended intervention to improve maternal and child health outcomes and increase access to facility-based deliveries. Barriers faced by women to use MWHs have been well documented, but there have been few fidelity studies on the implementation of MWHs.

**WHAT THIS STUDY ADDS**

- This study demonstrates the positive effect of community input for an intervention that meets community needs and outlines benchmarks to measure implementation fidelity in a meaningful and responsive way.

**HOW THIS STUDY MIGHT AFFECT RESEARCH, PRACTICE OR POLICY**

- The study outlines the implementation details of an MWH intervention and identifies pieces of the intervention perceived as critical for successful implementation, providing guidance to implementers seeking to replicate the intervention.

## Background

 Implementation fidelity—the degree to which an intervention is implemented as intended—is increasingly recognised as necessary to ensure that an intervention achieves its intended effects.^1^,^3^ Implementation effectiveness is influenced by the degree and quality with which a programme is implemented^4^ and is essential for efficacy and effectiveness. Efficacy refers to the degree to which a programme accomplishes the desired outcomes in favourable conditions, such as randomised control trials, while effectiveness is the ability to accomplish desired effects in real-world settings. Examining fidelity in efficacy studies helps determine what aspects of a programme are most important and needed to maintain the highest level of fidelity.^5 6^ Scoping reviews in 2015 and 2023 show the under-reporting of implementation fidelity to be an important gap in the field of implementation science and more data are needed to understand the link between implementation processes and outcomes.^7^,^9^

Implementation fidelity assessment is also important for effectiveness studies, such as randomised controlled trials of behavioural or systems-level interventions.^5^ Understanding how and why the implementation of an intervention deviated from its planned theory of change can help to better contextualise and interpret the eventual outcomes associated with the intervention and determine whether poor outcomes are due to the design, theory of change or implementation.^5 10^ Effectiveness studies without concurrent implementation fidelity assessment provide an incomplete picture of claims of cause and effect.^11 12^ Additionally, examining fidelity in effectiveness studies can inform decisions about replication, scalability and sustainability.^5 6^ As the literature on implementation fidelity continues to grow, examples of large, complex, systems-level intervention assessments can guide other programmes.

In a previously published paper, we examined the impact of a maternity waiting home (MWH) intervention using a quasi-experimental, cluster-controlled impact evaluation in rural Zambia. MWHs are lodgings near a health facility that allows women to stay near the health facility during the final days of pregnancy. We found a small but significant increase in access to health facility delivery among women living most remotely as well as improved secondary outcomes including MWH utilisation, exposure to health counselling and referral to higher-level care.^13 14^ However, recent studies suggest that poor implementation can negatively affect MWH acceptance and result in low utilisation by pregnant women.^15^,^17^

In Zambia, the government adopted MWHs as a strategy to increase facility-based deliveries.^18^ Here, as part of our concurrent implementation evaluation of an MHW intervention, we assessed the fidelity of implementation for the 10 health facilities randomly assigned to the intervention arm over the study period (October 2016 to July 2018). We compared the performance of MWHs to goals and assumptions detailed in our initial project plans to understand fidelity to the intervention and provide accountability to the community. Results will guide implementers seeking to replicate the MWH strategy and will contribute to the implementation fidelity literature in global health.

## Methods

### Study setting

The Maternity Waiting Homes Access in Zambia (MAHMAZ) project implemented a Core MWH Model at 10 health facilities in the rural districts of two provinces. MWH was built near a rural health facility providing routine preventive and curative services and uncomplicated deliveries. Women experiencing obstetrical complications were transferred to a district hospital. The 10 health facility catchment areas were similar in social economic status and ruralness and ranged in size from 5000 to 11 000 persons.^14^ The mean distance from the furthest village to the health facility in each catchment area was 23 km.^14^

Prior to the MAHMAZ project, the space allocated to pregnant women awaiting delivery within the study districts varied in quality and utilisation.^19^ Some facilities had community-constructed MWH structures: small mud-brick huts with limited infrastructure or amenities such as beds. Other facilities allowed women to sleep within the wards at night while other sites did not allow women to wait for delivery at all.

10 MWHs were constructed and implemented according to the standards set out by key stakeholders in the Core MWH Model.^20^,^22^ The standards are described in more detail below and in the published study protocol where additional information on site selection and randomisation can also be found.^23 24^

### Intervention description

The Core MWH Model, designed with community involvement, consisted of three main pillars—infrastructure, policies and linkages to care—each with its own set of components designed to be responsive to community standards for safety and comfort and to be culturally appropriate.^1720^,^22^ Key aspects of the Core MWH Model design included functional, lasting infrastructure with separate spaces for sleeping, recreation, sanitation, hygiene and cooking; amenities such as beds, mattresses, linens, mosquito nets and cooking utensils; systems of governance and management, including policies and management procedures to ensure the daily operations and long-term viability of the homes; check-ins from health facility staff to assure linkages to care; and health education classes provided by health facility staff and volunteers.

Governance committees were responsible for oversight of the daily operations of the MWHs, financial well-being and the long-term mission of the homes. They were implemented to optimise intervention delivery and make the MWHs more accountable to the communities. Governance committees included representation from villages in the health facility catchment area, community health workers, health facility staff and traditional leadership with a minimum of 50% female representation. More information on the MWH governance structures and their skills development workshops under MAHMAZ can be found elsewhere.^25^,^27^

The MWH governance committees engaged management units of 1–10 individuals whose members were responsible for the day-to-day management of the MWH, including intake and registration of women at the MWHs.

Study staff collaborated with local stakeholders and governance committees to develop standard operating procedures for the MWHs which describe the roles and responsibilities of the governance committee, management unit and users of the MWHs.

The MWHs opened following sensitisation meetings with traditional, religious and community leaders and members of the community. Health facility staff prioritised beds for pregnant women living>10 km from the health facility and encouraged the use of the MWHs for postnatal care (PNC).

Though our formative data suggested an average length of stay (ALOS) of 10 days,^22^ this was revised during implementation to adhere to Zambian government guidelines and WHO recommendations.^28^ Pregnant women were advised to arrive at the MWH approximately 2 weeks prior to their estimated delivery date to ensure that obstetrical complications could be identified early and managed or referred.^29^

Nine MWHs opened in September/October 2016. One MWH opened in March 2017.

### Study design

We collected longitudinal data from all sites from October 2016 to July 2018, considered the ‘implementation period’. Before the MWHs were constructed, evaluation questions and indicators to assess implementation fidelity were defined and documented in the project protocol^24^ and used as blueprints to guide intervention delivery.

### Theoretical framework

To guide our analysis, we used the conceptual framework of implementation fidelity (CFIF) by Carroll *et al*.^30^ In addition to measuring adherence to the intervention plan, CFIF also reviews potential moderators.^31^ Table 1 presents a description of the CFIF constructs for adherence to the plan (details of content, coverage, frequency and duration), potential moderators (quality of delivery, participant responsiveness and implementation strategies) and how we applied each construct to our intervention.

**Table 1:** Application of the conceptual framework of implementation fidelity (CFIF) constructs to the MAHMAZ intervention

CFIF construct	CFIF definition	Application to MAHMAZ	MAHMAZ indicator	Target	Data source
**Adherence: measurement of achievements against targets**					
Details of content	Description of the intervention	Whether MWHs met the core model components as described in the core model.	Proportion of months where each Core MWH Model component was met per site.	Meets the Core MWH Model components for at least 85% of the implementation period.	Core Model checklist
Coverage	Intervention reach	Whether MWHs served the target population of pregnant women living>10 km from the health facility.	Proportion of MWH users staying who lived in villages>10 km from the health facility.	Proportion of MWH users living remotely (≥10 km from health facility) matches or exceeds the proportion of the catchment area population living>10 km away.	MWH Register;Village GPS coordinates database
Frequency	Prescribed rate of intervention use or delivery	How the MWHs were used by the surrounding population?	AdmissionsNights stayedBed occupancy rate	80% bed occupancy	MWH Register
Duration	Prescribed period of intervention use or delivery	For how long women used the MWHs before and after their delivery?	Average length of stay for deliveryAverage length of stay for PNC	14-day average length of stay for deliveryNo target specific for PNC	MWH Register
**Potential moderators: subjective factors affecting outcomes**					
Quality of delivery	How well an intervention is delivered	Intervention users’ perceptions of quality of experience staying in MWHs.	Reported perceived quality overall and for multiple components of the Core MWH Model, including management and oversight of the MWH, cleanliness, friendliness of staff, access to a cooking area, perceived safety, boredom during stay and cultural appropriateness of the MWH.	No specific target	Experience survey with MWH users
Participant responsiveness	Participants perceptions about the value of intervention	Satisfaction and intent to return and recommend among MWH users.	Reported overall satisfaction with MWH stay. Reported intent to recommend MWH to a friend. Reported intent to return to MWH for future delivery or PNC stay.	No specific target	Experience survey with MWH users
Strategies to facilitate implementation	Approaches put in place to optimise or standardise intervention delivery	Composition of the governance committee.	Proportion of governance committee members who are women. Proportion of governance committees with traditional leadership, health facility staff and community health workers as members.	50% of governance committee members are women. Each governance committee has at least one member from traditional leadership, from the health facility staff and from the cadre of community health workers.	GovernanceCommittee Register

CFIF constructs and definitions adapted from Carroll C, Patterson M, Wood S, Booth A, Rick J, Balain S. A conceptual framework for implementation fidelity. Implementation Science. 2007; 2:1–9.

GPS, Geographical Positioning System; MAHMAZ, Maternity Waiting Homes Access in Zambia; MWH, maternity waiting homes; PNC, postnatal care.

For details of content, we define low adherence as meeting less than 85% of components in 85% of the observed months over the course of the implementation period. High adherence is defined as meeting 85% or more of the components in 85% or more of the observed months.

### Data collection

Data collection forms and methods are described below and in the implementation evaluation protocol.^24^

The Core Model Checklist (online supplemental file 1), quantitatively assessed the degree to which each element of the Core MWH Model was met at each site each month. Monitoring and evaluation (M&E) staff visited sites completed the checklist based on observations and also questioned facility staff and MWH managers about policies, procedures, finances, linkage to the health centre and services.

The MWH Register (online supplemental file 2), captured demographic information of each MWH user including name, a unique identifier assigned at check-in, health facility-issued identifier for each pregnancy (called the safe motherhood number), home village name, reason for stay (awaiting delivery or PNC visit) and arrival and discharge dates. Project M&E staff trained MWH managers to complete the register at check-in for each MWH user. M&E staff extracted data monthly.

Researchers conducted a quantitative experience survey (online supplemental file 3) with MWH users to capture perceptions and experiences during their stay. It included questions on the quality of intervention components, experience with Core MWH Model pillars (eg, availability of amenities for their use during their stay), satisfaction and intent to recommend or return for future use. The survey was completed monthly with women who had stayed at least three consecutive nights on the day of the survey. Three nights stay gave the women sufficient time to have a full experience of the MWH stay and experience the quality of care. Respondents were 15 years or older and had not previously completed the survey. Up to six surveys were conducted per month per MWH site.

An activities log (online supplemental file 4) was kept by MWH managers to track educational classes conducted. The register detailed the date, topic areas covered and number of attendees. M&E staff trained staff to complete the register each time health education was conducted. The log was extracted monthly.

A Governance Committee Register (online supplemental file 5), maintained by study site managers, included the basic demographics (age, sex and education) and affiliations (health facility staff, community health worker, traditional leadership) of governance committee members after initial selection and over time.

### Variables and analysis

The Core Model Checklist, experience surveys, MWH Register and activities log were collected using SurveyCTO Collect software (Dobility, Cambridge, Massachusetts, USA) on encrypted tablets. The electronic forms were sent to a secure server administered by SurveyCTO. Data cleaning and analyses were conducted in SAS V.9.4 (SAS Institute, Cary, North Carolina, USA).

### Core Model Checklist

We recorded the number of months that the Core Model Checklist was completed at each site (16–23 months; average 21 months). We first dichotomised variables under each core model components (infrastructure, equipment and supplies, n=21; policies, management and finance, n=8; linkages to the health system, n=6). Second, we created a variable to calculate the proportion of months each element of the Core MWH Model was present and in working condition. Third, we created six categories of adherence for each component: the element was present/working 100% of the month; 85–99.9% of the months; 70–84.9% of the months; 55–69.9% of the months; 40–54.9% of the months; below 40% of the months. In the lowest category, we had elements of components which were either not present or not working for<40% of the analysis period. To create the categories, we established a floor (40%) based on our observed data and then created even divisions to visualise the variations in the data. We assigned colours to each category and displayed the data using a heat map. Lastly, we dichotomised adherence to the main pillars of the Core MWH Model as high (≥85%) or low (<85%).

### MWH Register

For all MWH users, we calculated basic demographic information using means and SD or proportions. The reason for MWH use was designated as ‘awaiting delivery’ and ‘awaiting PNC visit’ (either immediately after delivery or within the following 6 weeks).

To estimate MWH-user travel distance, we used ArcGIS Online to calculate the most direct route between each health facility and the village centre, using the project database of catchment area villages. Distances were categorised as: <5 km, 5–9 km and≥10 km (rounded up from 9.5).

To calculate bed nights, we subtracted the admission date from the discharge date or the date of delivery if the discharge date was not available. In cases where the length of stay could not be calculated due to missing data (6.8%), a single imputation was used to model the length of stay. To calculate bed occupancy rate for all users at each MWH and overall, we divided the total bed nights for women awaiting delivery or staying for a PNC visit by the total number of possible bed nights. Possible bed nights were calculated as 10 beds (for women awaiting delivery) or 4 beds (for PNC women) multiplied by the total number of days in the implementation period.

To calculate the ALOS for each MWH and overall, we divided the total bed nights recorded for women awaiting delivery and for PNC women by the number of registered women in each category.

### Experience survey

Experience survey respondents were linked to demographics in the MWH Register. We calculated the same demographics for the subset of experience survey respondents as described above for all MWH users and compared demographics between the subset of experience survey respondents and the full MWH Register sample. We calculated the length of stay of each experience survey respondent at the time of the survey by subtracting the woman’s admission date from the date the experience survey was conducted.

We recorded the number of surveys completed at each MWH and the proportion of the responses for which each element of the checklist was present and in working condition. Respondents were asked to what degree components were satisfactory during their stay: for each component indicated, the responses indicated if there was (1) a major problem; (2) a minor problem; or (3) no problem. We created six categories of satisfaction for each component: The mean satisfaction score was 3.0 (meaning there were no months in which the component was not satisfactory); mean satisfaction score of 2.7–2.99; mean satisfaction score of 2.4–2.69; mean satisfaction score of 2.1–2.39; mean satisfaction score of 1.8–2.09; and mean satisfaction score of less than 1.8.

The experience survey also asked yes/no questions about participation in classes, intent to recommend the MWH and intent to return to the MWH. Here we also created six categories of adherence for each component: The respondents indicated satisfaction 100% of the months; 85–99.9% of the months; 70–84.9% of the months; 55–69.9% of the months; 40–54.9% or the months; below 40% of the months. We present categories as a heat map.

### Activities log

The activities log captured how many educational classes waiting women attended per week. The week of each date waiting women attended a class was assigned to the week of the year. Proportions were calculated to determine if classes were attended each week of the month and were coded as yes or no.

### Governance Committee Register

The mean number of committee members, mean number of executive committee members and proportion of governance committee members by sex and occupation were calculated using Microsoft Excel, overall and by site.

## Results

### Intervention user characteristics

Over 23 months, 3206 women used the MWHs across the 10 sites. On average MWH users were 24.4 years old, had attended primary school (grade 7) and had 3.3 previous pregnancies and 2.2 live births. The majority were married (85.7%) and were spread throughout the catchment area. Most MWH users stayed while awaiting delivery (83.4%) with an ALOS of 13.0 nights; 16.6% stayed for PNC and had an ALOS of 2.7 nights.

The subsample of experience survey respondents (n=448) was demographically similar to the main MWH users (online supplemental file 6), though a larger proportion came from more distant villages and had a longer ALOS of 21.3 nights. Experience survey respondents had stayed at the MWH on an average of 12.8 nights.

### Adherence to details of content

**Figure 1:**
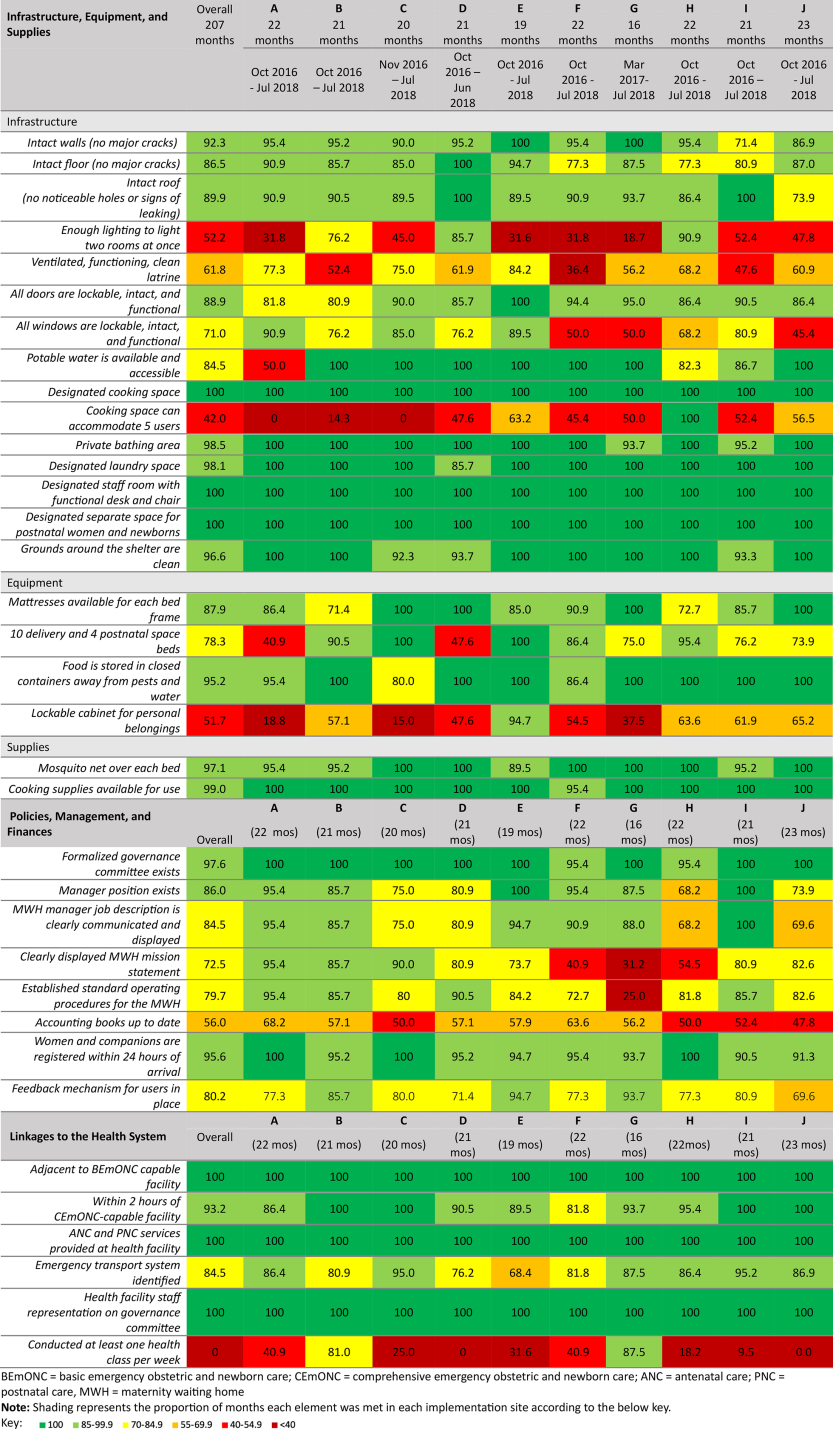
Heat map showing the adherence to details of the content of the Core MWH Model.

Figure 1 presents adherence to each element of the Core MWH Model for each site and overall. As indicated in light and dark green, there were generally high levels of adherence with adherence over 85% of the months for many elements. Within the infrastructure, equipment and supplies pillar, the lowest adherence was for sufficient lighting in the MWH and cooking spaces. Lower adherence was also experienced for lockable cabinets for personal belongings due to lost keys.

Under policies, management and finances, the mission statements were not always displayed in a location visible to users and standard operating procedures were not always available to the assessor; at times these were displayed in the locked office or storage room and therefore rated as non-adherent.

Within the linkages to the health system pillar, all MWHs were constructed near a health facility that was within 2 hours from a comprehensive emergency obstetrical and newborn site, though five sites still reported challenges with travel time. Seven MWHs held an average of at least one class per week over the implementation period.

Emergency transport systems identified by health facility staff for transport of obstetrical cases to higher level care included district ambulances (330, 98%), local taxis (4, 1%) and private transport (2, 0.6%).

Overall, 70% of sites exhibited high adherence to the Core MWH Model over the implementation period. Table 2 depicts the proportion of sites with low (<85%) and high (>85%) adherence to the main pillars of the Core MWH Model and overall.

**Table 2:** Summary of the adherence to details of content within the Core MWH Model pillars

Core MWH Model pillars	Adherence to details of content	
	Proportion of sites (out of 10) with low adherence (less than 85%)	Proportion of sites (out of 10) with high adherence (85% or above)
Infrastructure, equipment and supplies	40% sites (site A, F, G, I)	60% sites (site B, C, D, E, H, J)
Policies, management and finances	70% sites (site C, D, F, G, H, I, J)	30% sites (site A, B, E)
Linkages and services	30% sites (site D, E, F)	70% sites (site A, B, C, G, H, I, J)
Overall	30% sites (site F, I, J)	70% sites (site A, B, C, D, E, G, H)

MWH, maternity waiting home.

### Coverage

**Figure 2:**
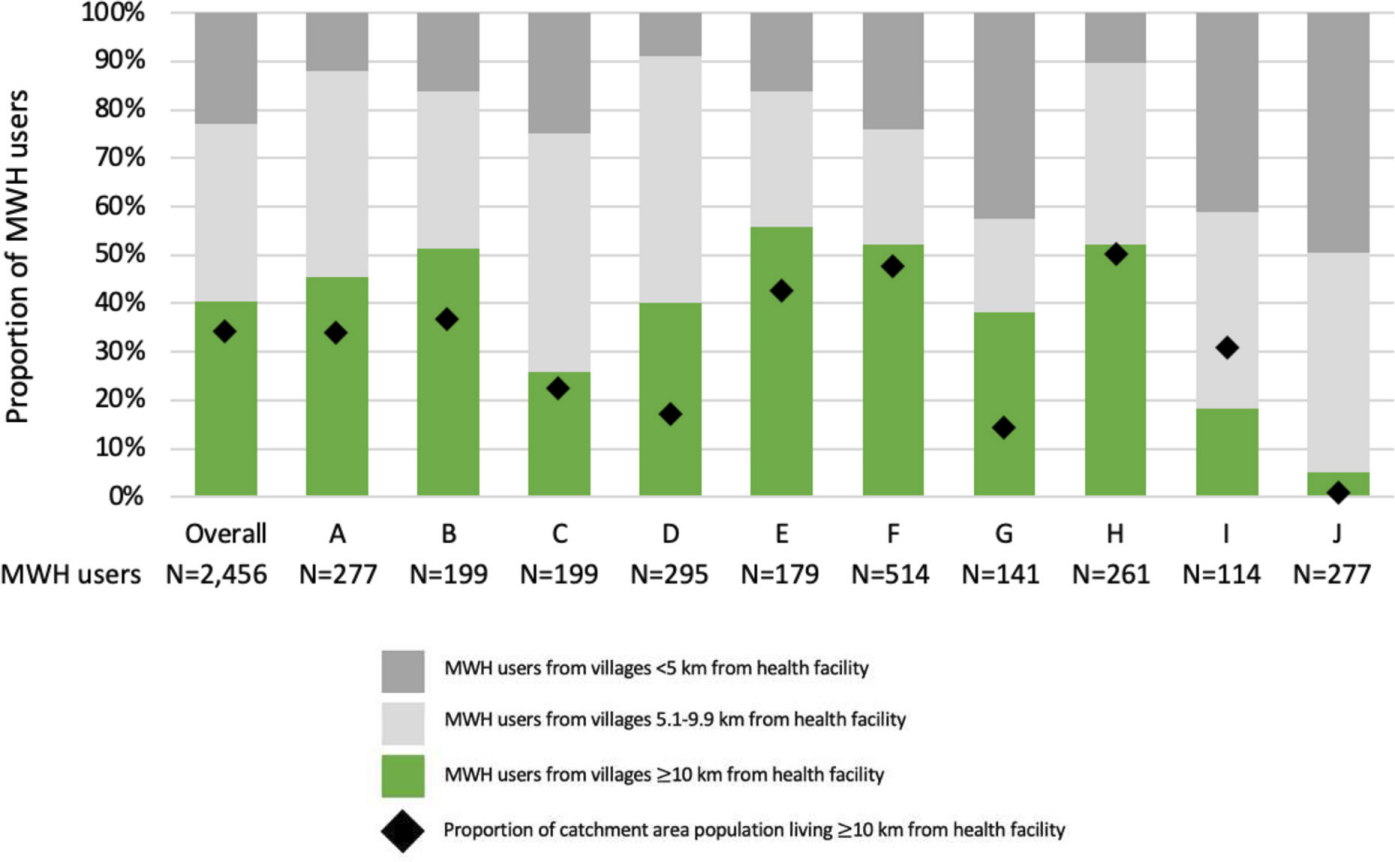
Intervention coverage—Proportion of MWH stays for delivery by a distance of user’s village to the rural health centre compared with the proportion of catchment area population living 10 km or more away. Note: 217 MWH users could not be linked to known villages. MWH, maternity waiting home.

Figure 2 shows the proportion of MWH stays for delivery by distance from the user’s village to the rural health centre compared with the proportion of the catchment area population living 10 km or more away. Generally, the proportion of women staying at an MWH who come from a village ≥10 km from a health facility was consistent with the proportion of the population living ≥10 km from that facility (figure 2). The site I did not have users from>10 km proportionates to the surrounding population. Site J had a very low proportion of population living>10 km and few MWH stays were among users from villages ≥10 km; 50% of users lived within 5 km of the health facility.

### Duration and frequency

Over the implementation period, 3206 women used the MWH for 36 281 nights. Site J had the fewest users (n=122) while Site F had the most (n=586). The overall occupancy rate was 52% for the 10 sites, ranging from 22% (Site I) to 134% (Site F). An occupancy rate of over 100% was possible using extra mattresses placed on the floor. The overall occupancy rate and the occupancy rate for 9 of 10 sites was below the target of 80%. The overall ALOS for users awaiting delivery was 13.0 nights, ranging from 8.4 to 16.0. Eight sites had ALOS below the target of 14 nights. Table 3 summarises the MWH stay statistics for all users. Online supplemental file 7 provides facility-level detail.

**Table 3:** Intervention duration and frequency

	Target	Overall
Theoretical framework construct: frequency		
Total admissions	NA	3206
Total nights stayed	NA	36 281
Occupancy rate	80%	52%
Theoretical framework construct: duration		
Average length of stay (nights) for delivery stays	14.0	13.0
Average length of stay (nights) for postnatal care stays	NA	2.7

MWH users perceived high quality and general satisfaction at each site and overall. The only domains with low perceived quality included access to the cooking area and participation in a class, both connected to low adherence to details of content. 99.3% of respondents reported an intent to recommend the MWH to a friend and 96.2% intended to return for a future delivery. 74.6% of MWH users reported an intent to return for a PNC visit. Online supplemental file 8 shows a heat map depicting MWH users’ perceptions and satisfaction with key elements of the Core MWH model, by site and overall.

### Moderator: strategies to facilitate implementation

MWH governance committees included 9.3 members on average (online supplemental file 9). Approximately 40% of members were female (range: 29–50%); 31% were women of reproductive age (the target population for the intervention). Only three sites reached 50% female membership on the governance committee. All committees had a health facility staff member and seven sites had traditional leadership representatives. Most members were community-based volunteers. Approximately half of the committee members held executive committee positions, of which 42% were female. Site I had no female members on the executive committee.

## Discussion

This fidelity evaluation of an MWH project in rural Zambia contributes to filling the evidence–practice gap for the WHO recommendation of implementing MWHs and strengthens the growing literature on implementation fidelity studies in global health overall.^32^ When evaluating the implementation fidelity of our community-informed MWH intervention,^20^,^22^ we found reasonably high fidelity when examined through the lens of the CFIF. Adherence to details of content was generally high across all pillars of the Core MWH Model with some notably low ratings in several components. Access to electricity in rural Zambia is as low as 4.3% of the population, resulting in increased use of alternative energy sources such as solar.^33^ All the MWHs were set up for connection to the national electric grid to comply with the mandate of the Rural Electrification Authority; however, few sites were connected during the implementation period. Sites that were not connected relied on solar-powered bulbs which did not always provide adequate lighting, resulting in the poor measures of fidelity observed under the infrastructure, equipment and supplies pillar. Additionally, lockable cabinets were generally not available and the cooking space could not accommodate five individuals at once. Despite these low ratings, women generally reported feeling safe during their MWH stay, an indication that the standards of safety implemented by the programme met the minimum community requirements. The MWH users’ perceptions and satisfaction with key elements of the Core MWH Model underpins the importance of community involvement in project design for cultural appropriateness.^34^ The adaptations and deviations from the initial implementation plans were not surprising and are reflected in our observed results.

In the policies, management and finance pillar, the accounting books were rarely up to date. Skills training in financial management was provided to members of the governance committee to enable them to perform tasks necessary for supervision responsibilities.^27^

In the linkages to the health system pillar, there was very low adherence to attending at least one health class per week. Our evaluation noted that not all sites offered the recommended number of classes per week and it was up to the women to attend the classes. The low frequency of classes and the optional participation may explain these results. Further consultation with waiting women may have elicited factors contributing to the low rating, but we did not do this.

As a measure of equity, in its intention to reach the most remote living women, the MWH’s coverage was good, as indicated by the proportion of women staying at an MWH who came from a village ≥10 km from a health facility being consistent with the population share. Potentially more equitable access to the intervention was observed for sites A, B and D where a higher proportion of women living ≥10 km away used the MWH compared with those close to the homes. This is consistent with findings from the impact evaluation which found that in the overall study, improved MWHs were associated with more than twice the odds of using an MWH while awaiting delivery and ultimately increased odds of delivering at a facility among women living in villages ≥10 km from the health facility.^14^ In addition to addressing the distance barrier to accessing health facilities, by implementing the components of the infrastructure, equipment and supplies pillar, the Core MWH Model also addressed social and economic barriers to using MWHs.^35 36^ This integrated approach to increasing access to MWHs was a critical piece of implementing and generating equitable access to the Core MWH Model.

In terms of duration, the ALOS of 13 days was congruent with the plan based on government guidelines and while in some sites the average was less than 10 days, it still suggests women arrived well before labour. This allows sufficient time for health facility staff to observe and better manage obstetrical complications, a major challenge when someone arrives in active labour.^29^ The bed occupancy rate, however, our fidelity measure for frequency, varied substantially across sites. With an occupancy rate of 134%, site F was a large catchment area and had the highest number of births at the rural health centre (56, compared with approximately 20 at most others). The MWH at site F was not large enough to accommodate the population and this was reflected in the high occupancy rate. Despite the variations in the occupancy rate, the overall perceived quality and reported satisfaction by MWHs were high. A previous analysis that also noted variability in occupancy rates observed that this may be unavoidable in rural, less densely populated areas and that updated, reliable data for planning was needed.^37^ Researchers and implementers seeking to roll out MWH interventions should consider site-specific bed capacity adaptations for the delivery of MWHs responsive to local contexts.

The final CFIF moderator, strategies to facilitate implementation, was measured by the composition of the MWH governance committee members. While about 40% of committee members were female, only three sites met the target of 50% female membership. The recommendation that 50% of females comprise the governance committees was generated during early stakeholder engagement meetings with traditional chiefs, health facility staff, ministry officials, local community leaders and reproductive-aged women. Historically in Zambia, men hold leadership roles; advising a more balanced leadership team by gender was an intentional attempt to empower women in the communities. Female representation, in fact, got worse over time with fewer women elected at the annual committee elections than at startup,^25^ but there was a higher proportion of women in key executive committee roles (42%) compared with the general membership which is encouraging for gender equity and inclusion. Notably, all sites had a governance committee member from the health facility which was critical to the linkages to the health system component of the Core MWH Model and 70% of sites had membership by traditional leadership, a hypothesised critical element of community ownership and sustainability. In previously published work, Fontanet *et al* discussed community ownership of the Core MWH Model and the critical roles and responsibilities of the governance committees for long-term success of the MWHs.^26^ Overall, we found early and continuous engagement of a broad range of stakeholders to be instrumental to our high implementation fidelity and participant responsiveness measures.^38^ Investing in a systematic and broad stakeholder engagement strategy can be costly and time-consuming, but we strongly recommend implementers plan for it and that donors support the cost and time as part of their investments.

We anticipated some variability in implementation and as such variability around the Core MWH Model checklist components and participant experience. We found that informal governance was sometimes preferred over formal procedures. In fact, in many countries, informal interpersonal communications are a valued tool for promoting answerability and enforcement.^39 40^ Informal governance can complement formal governance by enhancing information sharing and developing innovative solutions. For example, we set up a formal feedback mechanism through a suggestion box placed at each MWH for users to write and deposit anonymous feedback during their stay. However, users preferred to provide informal feedback directly to managers, governance committees or health facility staff. We hypothesise this could be because of predominantly oral culture and low literacy levels. The intention of a visible mission statement and feedback mechanisms was to ensure women were aware of the MWH purpose and felt empowered to modify operations and hold the governance committees accountable. Despite the formal system not being implemented as intended, it is encouraging that most sites still employed some feedback mechanisms for accountability. This is similar to findings in South Africa where informal relationships of trust allow people to share experiences related to maternal care in a spontaneous manner, facilitating accountability through negotiated agreements, unofficial action or consensus-building strategies.^41^

While we were not powered to determine the effects of individual Core MWH Model components on outcomes of MWH use or facility-based delivery, the fidelity of implementation was within reason and gives context and confidence to the results observed in the impact study.^14^ The community input was instrumental in the success of the intervention, both in its design to meet community needs and in the standards outlined to measure implementation compliance in a meaningful and responsive way.

MWHs are complex, system-level interventions that depend on the overall structure of the health system and on key factors that both motivate and enable the end user to use the MWH. This complexity is one key reason for the intensive investments we made in our stakeholder engagement strategy. Despite this, however, our findings here and elsewhere consistently suggest that a one-size-fits-all model may not be the appropriate approach.^37^ Considering this, it may be useful to not only allow for different contextually appropriate MWH models but to also capture the site-specific adaptations systematically to better understand the fidelity-adaptation balance, as proposed by Perez *et al* and others.^2 42^

### Limitations

This analysis of multiple data sources to understand implementation fidelity allowed us to understand fidelity against the project’s initial targets. However, there are several limitations. First, data were only collected for an average of 21 months per site while the homes were operating, so we are limited in understanding longer-term fidelity. Additionally, the role of the MAHMAZ project staff was to coach the local governance committees through implementation. Given that staff on the same project worked to coach or collect the data, there was potential for both researcher and respondent biases. To mitigate this, data collection forms were purely quantitative and data collectors were trained to be as objective as possible. Additionally, because the inclusion criteria for the experience surveys required women to have stayed at least three nights, we had no respondents who were staying for PNC. Therefore, we cannot speak to the implementation fidelity or quality from the perspective of those who stayed only for PNC. Lastly, the intervention did not include an assessment of the quality of educational classes which could directly influence participation.

## Conclusion

This study found relatively high fidelity to implementation, as measured against benchmarks set by the community and other key stakeholders during the development phase but that fidelity varied by site and depended on contextual factors including catchment size. Fidelity measures are critical for understanding and interpreting outcome results, may inform site-specific adaptations and contribute to filling gaps in evidence to practice for MWHs and fidelity studies in global health more broadly.

## Supplementary material

10.1136/bmjph-2024-001215 online supplemental file 1

10.1136/bmjph-2024-001215 online supplemental file 2

10.1136/bmjph-2024-001215 online supplemental file 3

10.1136/bmjph-2024-001215 online supplemental file 4

10.1136/bmjph-2024-001215 online supplemental file 5

10.1136/bmjph-2024-001215 online supplemental file 6

10.1136/bmjph-2024-001215 online supplemental file 7

10.1136/bmjph-2024-001215 online supplemental file 8

10.1136/bmjph-2024-001215 online supplemental file 9

## Data Availability

Data are available in a public, open access repository.
