# Supplementary material for: Implementation fidelity of a multisite maternity waiting homes programme in rural Zambia: application of the conceptual framework for implementation fidelity to a complex, hybrid-design study
Source: BMJ Public Health. 2025 Jan 16;3(1):e001215. doi: 10.1136/bmjph-2024-001215 (PMC11812881; doi:10.1136/bmjph-2024-001215)
Supplement: online supplemental file 2 [file bmjph-3-1-s002.pdf]

District:________________________

Province: ______________________

Health Facility ID: ______________________

Maternity Homes Alliance
Mothers' Shelter Register

A	B	C	D	E	F	G	H	I	J	K
**MS Registration number**<br><br>Write the MS registration number here and place a sticker with the same number on the woman's ANC card	**Safe Motherhood Number**<br><br>(Copy from ANC card)<br><br>*Write "unavailable" if SMN unavailable*	**Date of arrival to MS for this visit**<br><br>(dd/mm/yy)	**Reason for MS stay for this visit**<br><br>(1) 1st ANC visit,<br>(2) Other ANC visit,<br>(3) While awaiting delivery<br>(4) After discharge from health facility/ immediately after delivery<br>(5) 3 day post natal visit<br>(6) 7-14 days post natal visit<br>(7) 6 weeks post natal visit	**Woman's full name**<br>(First name, Surname)	**Marital Status**<br><br>(1) Married/Cohabiting<br>(2) Divorced<br>(3) Separated<br>(4) Widowed<br>(5) Never-married	**Village name**	**Origin code**<br><br>**HF only:**<br>(1) From within 12km, within the catchment area<br>(2) From more than 12km, within the catchment area<br>**Both HC and Hospital:**<br>(3) From within district but outside catchment area<br>(4) From outside district<br>(5) From outside Zambia<br>(97) Unknown	**Date discharged from MS/ Transferred to HF**<br><br>(dd/mm/yy)	**Reason for Discharge / Transfer**<br><br>(1) Transferred to HF for delivery or complications,<br>(2) Returned home without delivering,<br>(3) Returned home after ANC visit<br>(4) Returned home after postpartum stay<br>(96) Other. If other, specify	**Date of delivery**<br><br>If returning from facility after delivery<br><br>(dd/mm/yy)<br><br>***If woman did not return to the MS after delivery, check with health facility staff for delivery date and enter here**
__-_____-___-___-_____	_____-___-_______-___	/ /						/ /		/ /
__-_____-___-___-_____	_____-___-_______-___	/ /						/ /		/ /
__-_____-___-___-_____	_____-___-_______-___	/ /						/ /		/ /
__-_____-___-___-_____	_____-___-_______-___	/ /						/ /		/ /
__-_____-___-___-_____	_____-___-_______-___	/ /						/ /		/ /
__-_____-___-___-_____	_____-___-_______-___	/ /						/ /		/ /
__-_____-___-___-_____	_____-___-_______-___	/ /						/ /		/ /
__-_____-___-___-_____	_____-___-_______-___	/ /						/ /		/ /
__-_____-___-___-_____	_____-___-_______-___	/ /						/ /		/ /
__-_____-___-___-_____	_____-___-_______-___	/ /						/ /		/ /
__-_____-___-___-_____	_____-___-_______-___	/ /						/ /		/ /
__-_____-___-___-_____	_____-___-_______-___	/ /						/ /		/ /

District:________________________

Province: _______________________

Maternity Homes Alliance
Mothers' Shelter Register

Health Facility ID: ______________________

L	M	N	O	P	Q	R		S	T	U	V		W	X
**Age at last birthday**	**Highest grade completed**<br>Write 00 for no education	**EDD**<br>(dd/mm/yy)	**Gravida**<br>Copy from ANC card if available<br>If not available, ask the woman 'how many times have you been pregnant?' and write the number here	**Parity**<br>Copy from ANC Card if available<br>If not available, ask the woman 'how many deliveries >20weeks/ 5 months in pregnancy have you had?' and write the number here	**Number of previous still births**<br>Ask woman 'how many stillbirths have you had from previous pregnancies?' and write the number here	**Number of companions**		**Companions**<br>Provide response for each companion:<br>(1) Mother<br>(2) Mother-i- law<br>(3) Aunty<br>(4) Grandmother,<br>(5) Sister<br>(6) Child<br>(7) Husband<br>(96) Other.<br>If other, specify	**Heard of MS from:**<br>*(select all that apply)*<br>(1) Chief<br>(2) Headman<br>(3) Health care worker<br>(4) SMAG<br>(5) Traditional birth attendant<br>(6) Family member<br>(7) Another mother<br>(8) Other community member<br>(9) Radio<br>(96) Other<br>If other, specify.	**Transport method used**<br>(1) Walking<br>(2) Bicycle<br>(3) Carried in wheelbarrow<br>(4) Animal drawn cart<br>(5) Taxi<br>(6) Car<br>(7) Motorcycle<br>(8) Ambulance<br>(96) Other<br>If other, specify.	**Travel time from home to mothers' shelter**		**Participated in Experience Survey?**<br>Tick here if participated in experience survey	**Comments**
						**F**	**M**				**Hr**	**Min**		
__ __		/ /												
__ __		/ /												
__ __		/ /												
__ __		/ /												
__ __		/ /												
__ __		/ /												
__ __		/ /												
__ __		/ /												
__ __		/ /												
__ __		/ /												
__ __		/ /												
__ __		/ /												
