# Supplementary material for: Implementation fidelity of a multisite maternity waiting homes programme in rural Zambia: application of the conceptual framework for implementation fidelity to a complex, hybrid-design study
Source: BMJ Public Health. 2025 Jan 16;3(1):e001215. doi: 10.1136/bmjph-2024-001215 (PMC11812881; doi:10.1136/bmjph-2024-001215)
Supplement: online supplemental file 3 [file bmjph-3-1-s003.pdf]

Facility ID: __ __ __ __ __ __
Today's Month/Year (MM/YYYY): ___ ___/ ___ ___ ___ ___

Mothers' Shelter Experience Survey

Maternity Homes Alliance

Instructions
Please complete this survey ***only with a respondent who has stayed at the mothers' shelter or the designated space for waiting mothers before or after delivering for at least 3 nights in a row.***

Province: ___________________________

District: ____________________________

Health Facility Name: __________________________________

Health Facility ID: ☐☐☐☐☐☐

Date of interview: ☐☐ ☐☐ ☐☐☐☐

D D M M Y Y Y Y

Data Collector Name: ________________

Short screen:

No	Question	Response	Instructions
SS1.	How old were you at your last birthday?	____ ____	**If <15, end the survey.**<br>**If <18 but >15, get assent from participant and consent from guardian.**
SS2.	Have you slept here (mothers' shelter/existing structure) at least 3 nights in a row?	Yes (1)<br>No (0)	**If (0), end the survey**
SS3.	What is the purpose of your current stay?	ANC (1)<br>Waiting to deliver (2)<br>PNC (3)<br>Other (4)	If (1) or (2), proceed to SS5.<br>If (3), continue to SS4.<br>**If (4), end the survey**
SS4.	If purpose of current stay is PNC, how old is your baby?	_____ Days (1)<br>_____ Weeks (2)<br>Less than 1 day old (<24 hours) (3)	**If baby is more than 6 weeks old, end the survey**
SS5.	Have you previously participated in an experience survey for this kind	Yes (1)<br>No (0)	If (0), proceed to consent.<br>**If (1), end the survey.**

	of stay (respondents' response in Q3)		

Was written informed consent obtained, and consent form labeled with the correct ID number?

YES ___ (proceed with interview)

NO ___ (**STOP!** Thank the participant for their time but **do not proceed** with the interview)

Start Time: __________ **Finish Time: _____________**

Facility ID: __ __ __ __ __ __
Today's Month/Year (MM/YYYY): ___ ___/ ___ ___ ___ ___

General Questions About Mothers' Shelter Stay

MSID: ☐☐☐☐☐☐☐☐☐☐☐☐☐
write as it appears in the MS register

SMN register:
write as it appears in the MS register

SMN ANC card:
write as it appears on the ANC card

Do these numbers match?
If no, make the appropriate changes after the survey.

Instructions: Read the following to the respondent: "I will ask you general questions about your stay here."

#	Question	Response	
**How many women are staying at the MS today?**			
**1.**	How many nights have you slept here during this stay?	____________________	
1a	How many companions are waiting with you during this stay?	____________	If <=1, skip to 2.
1b	If more than one companion, where are they all sleeping at night?		
**2.**	Were you told about any rules or procedures within one day (24 hours) of arriving?	(1) Yes<br>(0) No<br>(97) Don't know	
**3.**	Did a manager/caretaker for this shelter visit yesterday?	(1) Yes<br>(0) No<br>(96)NA, no manager/ caretaker<br>(97) Don't know	
**4.**	Did a health care worker from the health facility check in on you yesterday?	(1) Yes<br>(0) No<br>(97) Don't know	
**5.**	Is there a system in place for you to share your opinion about the mothers' shelter and your experience here?	(1) Yes<br>(0) No<br>(97) Don't know	If (0) or (97), skip to 6
**5a.**	Please tell me any methods that are in place to learn about your opinions of the shelter.<br><br>*Select all that apply.*	(1) Suggestion box<br>(2) Client survey form (separate from this one)<br>(3) Official meeting with community leaders	

Facility ID: __ __ __ __ __ __
Today's Month/Year (MM/YYYY): ___ ___/ ___ ___ ___ ___

		(4) Informal discussion with community<br>(5) Letters<br>(6) Other (specify):___________	
**6.**	Did you sleep under a mosquito net last night?	(1) Yes<br>(0) No	
6a.	If No, why not?	(1) No mosquito net<br>(2) Chose not to sleep in net<br>(3) Other (specify): ___________	
**7.**	Was there a bed with a mattress or a mattress on the floor available for you every night during your stay?	(1) Yes<br>(0) No	
**8.**	Have you shared (overnight) a bed or mattress with another adult at any time during your stay?	(1) Yes<br>(0) No	
**8a.**	If yes, was this adult:	(1) Your companion<br>(2) Another waiting mother<br>(3) Other<br>(96) Not applicable	If (1), (2), or (96) skip to 9
**8b.**	If Other, please specify:	____________________	
**9.**	When you went to get water last, were you able to access it?	(1) Yes<br>(0) No<br>(96) NA/Did not go to get water	
**10.**	Do mothers have access to a private bathing area?	○ Yes, a separate area (1)<br>○ Yes, but bathing from the latrines (2)<br>○ No (0)<br>○ N/A (96)	If (0) or (96), Skip to 10b
**10a**	In general, how clean is the bathing area?	○ Sufficiently clean (1)<br>○ Clean but needs improvement (2)<br>○ Not clean (3)	
**10b**	Do mothers have access to latrines?	○ Yes (1)<br>○ No (0)<br>○ N/A (96)	If (0) or (96), Skip to 11
**10c**	In general, how clean are the latrines?	○ Sufficiently clean (1)<br>○ Clean but needs improvement (2)<br>○ Not clean (3)	

Facility ID: __ __ __ __ __ __
Today's Month/Year (MM/YYYY): ___ ___/ ___ ___ ___ ___

**11.**	Do you have access to a washing area?	(1) Yes<br>(0) No	
**12.**	Did you cook here yesterday?	(1) Yes<br>(0) No	If (1), skip to 13
**12a.**	If No, why not?<br><br>*Select all that apply*	(1) No cooking area<br>(2) Cooking area not accessible<br>(3) No pots/pans/utensils<br>(4) Not enough fuel<br>(5) Not enough food<br>(6) Food provided/shared/did not need to cook<br>(7) Other	
**12b.**	If Other (7) in 12a, please specify:	______________________	
**13.**	Did you go to bed hungry last night?	(1) Yes<br>(0) No	
**14.**	Did the shelter have a source of light after the sun went down last night (e.g. candle, kerosene, electricity)?	(1) Yes<br>(0) No<br>(97) Don't know	
**15.**	In general, do you feel safe staying here?	(1) Yes<br>(0) No	
**15a.**	If no, why?	________________________	
**16.**	Did you lock your personal belongings in a cabinet yesterday?	(1) Yes<br>(0) No	
**16a.**	If no, why?	(1) Didn't want/ care to<br>(2) No lock available<br>(3) No key available<br>(4) Broken cabinet<br>(5) Other (specify):___________	
**17.**	Have any of your personal belongings gone missing during your stay?	(1) Yes<br>(0) No	
**18.**	How often, if ever, have you felt bored during your stay?<br><br>*Read responses to respondent*	(1) All the time<br>(2) Often<br>(3) Sometimes<br>(4) Rarely<br>(5) Never	
**19.**	Have you attended any education class during your stay?	(1) Yes<br>(0) No	If No, skip to 20
**19a.**	If Yes, how many classes have you attended?	_________________	

**20.**	If you become pregnant again, would you stay here before delivery?	(1) Yes<br>(0) No<br>(97) Don't know
**20a.**	If you have another baby, would you return here to stay for a postnatal visit?	(1) Yes<br>(0) No<br>(97) Don't know
**21.**	Would you recommend staying here to a friend or relative?	(1) Yes<br>(0) No<br>(97) Don't know
**22.**	In general, how satisfied are you with your stay here?	(1) Very satisfied<br>(2) More or less satisfied<br>(3) Not satisfied

Now I'm going to ask you some common problems women face at mothers' shelters while staying there. As I mention each one, please tell me whether any of these were a problem for you during your stay here, and if so, if they were a major or minor problem, or no problem for you.		Major problem (1)	Minor problem (2)	No problem (3)	Undecided (97)
**23.**	Overall quality of the shelter?				
**24.**	Management and oversight at the shelter?				
**25.**	Cleanliness of the shelter?				
**26.**	Presence of the staff?				
**27.**	Friendliness of the staff while staying at the shelter?				
**28.**	Access to cooking area?				
**29.**	Crowdedness of the shelter?				
**30.**	Safety while staying at the shelter?				
**31.**	Boredom while staying at the shelter?				
**32.**	Cultural appropriateness of the shelter?				

Facility ID: __ __ __ __ __ __
Today's Month/Year (MM/YYYY): ___ ___/ ___ ___ ___ ___

Questions about Costs

Instructions: Read the following to the respondent: "I will now ask you questions about what you would be doing if you were not staying here."

**33.**	If you were not here, what activities in your home would you be doing today?<br><br>*Select all that apply*	(1) Caring for children<br>(2) Caring for parents/relatives<br>(3) Caring for husband<br>(4) Cooking for family<br>(5) Caring for house/Cleaning<br>(6) Laundry<br>(7) Caring for animals/livestock/chickens<br>(8) Farming/gardening<br>(9) Gathering water/firewood<br>(10) Food processing (e.g. grinding maize)<br>(11) Other<br>(12) Nothing	If (12), skip to 34
**33a.**	If Other, please specify:	___________________________________	
**33ai**	Are you doing any of these activities here today?	(1) Yes<br>(0) No	
**33b.**	If yes, what are these activities?<br><br>*Select all that apply*	(1) Caring for children<br>(2) Caring for parents/relatives<br>(3) Caring for husband<br>(4) Cooking<br>(5) Caring for house/Cleaning<br>(6) Laundry<br>(7) Caring for animals/livestock/chickens<br>(8) Farming/gardening<br>(9) Gathering water/firewood<br>(10) Food processing (e.g. grinding maize)<br>(11) Other	
**33c.**	If Other, please specify:	___________________________________	
**34.**	If you were not here, what activities outside your home would you be doing today?<br><br>*Select all that apply*	(1) Piecework<br>(2) Food processing (e.g. grinding maize)<br>(3) Shopping at market<br>(4) Selling at Market/Kiosks<br>(5) Community work<br>(6) Other employment<br>(7) Other<br>(8) Nothing	If (8), skip to 35a
**34a.**	If Other, please specify:	___________________________________	

Facility ID: __ __ __ __ __ __
Today's Month/Year (MM/YYYY): ___ ___/ ___ ___ ___ ___

**34ai**	Are you doing any of these activities here today?	(1) Yes<br>(0) No	
**34b.**	If yes, what are these activities?<br><br>*Select all that apply*	(1) Piecework<br>(2) Food processing (e.g. grinding maize)<br>(3) Shopping at market<br>(4) Selling at Market/Kiosks<br>(5) Community work<br>(6) Other employment<br>(7) Other	
**34c.**	If Other, please specify:		
**INSTRUCTIONS:** Look back at Question 1 to see how many nights the respondent has stayed at the MS so far.<br>***READ: "You have been here for (X number of) nights. Now I'd like to ask you about costs associated with your stay."***			
**35a.**	Did you set aside money for your delivery?	(1) Yes<br>(0) No	
**35b.**	About how much did you spend in preparation for your delivery (such as supplies, clothes, and other items for you or the baby, but not including transport)?	_____________ Kwacha<br><br>If none, enter "0"	
**36.**	Did you pay someone to bring you here to the shelter?	(1) Yes<br>(0) No	If No, skip to 35
**36a.**	If Yes, how much did you pay? If in-kind, estimate the value.	_____________ Kwacha	
**37.**	About how much have you had to spend during your stay here so far (for items such as food, accommodation, provider/health center fees, informal payments, tips, in-kind resources, drugs, diagnostic test, other fees)?<br><br>*Write down the amounts the woman says in your field notebook and check if the total amount of the items she said adds up correctly.*	_____________ Kwacha<br><br>If none, enter "0"	
**38.**	Have you paid to stay in this MS so far?	(4) Yes<br>(0) No [skip to 39]	
**38a.**	If yes, how much?	_________________Kwacha<br>[skip to 39a]	
**39.**	If you had to pay, would you stay here?	(1) Yes<br>(0) No [skip to 40]	

Facility ID: __ __ __ __ __ __
Today's Month/Year (MM/YYYY): ___ ___/ ___ ___ ___ ___

**39a.**	How much maximum would you be willing to spend each night (for the same experience as you have had)?	_______________Kwacha/night	
**40.**	Would you be interested in telling us more about your experience in the future?	(1) Yes<br>(0) No	If No, end survey
**41a.**	If Yes, in what village do you stay?		
**41b.**	If Yes, what is the best way to contact you?		
**42.**	Do you have any final comments to share with us about your stay here?		

Instructions: Read the following to the respondent, *"Thank you sincerely for your time. We have completed this survey and are grateful for your help as we work to develop or improve on the mothers' shelters in your community."*

End the survey.

Reminder to interviewer: If the SMN from the register did not match the SMN on the woman's ANC card, correct the SMN in the register based on the SMN on the ANC card.
