# Supplementary material for: Implementation fidelity of a multisite maternity waiting homes programme in rural Zambia: application of the conceptual framework for implementation fidelity to a complex, hybrid-design study
Source: BMJ Public Health. 2025 Jan 16;3(1):e001215. doi: 10.1136/bmjph-2024-001215 (PMC11812881; doi:10.1136/bmjph-2024-001215)
Supplement: online supplemental file 4 [file bmjph-3-1-s004.pdf]

Mothers' Shelter Activities Log

Form F

Facility ID: _______
Facility Name: ______________________
Province: ________________
District: _________________

Today's Date (dd/mm/yyyy): ___/___/_____
Data Calendar (mm/yyyy): ___/_____
Collector Name: _________________

A			B1	B2	B3	B4	B5	B6	B7	C1	C2	C3	D	E	F	G
Date			Type of Activity							Number of People Attending			Facilitator's Name (First name, Surname)	Facilitators title	Description of Activity / Remarks	Facilitator's signature
dd	mm	yy	Family Planning Class	Breast-feeding and IYCF Class	Newborn Danger Signs / Baby Care Class	ANC/ PNC Class	Other Class	Income Generating Activity / Class	Other Activity	Women Staying at Mothers' Shelter	Others	Total people in attendance				
— —	— —	— —														
— —	— —	— —														
— —	— —	— —														
— —	— —	— —														
— —	— —	— —														
— —	— —	— —														
— —	— —	— —														
— —	— —	— —														
— —	— —	— —														
