# Supplementary material for: Implementation fidelity of a multisite maternity waiting homes programme in rural Zambia: application of the conceptual framework for implementation fidelity to a complex, hybrid-design study
Source: BMJ Public Health. 2025 Jan 16;3(1):e001215. doi: 10.1136/bmjph-2024-001215 (PMC11812881; doi:10.1136/bmjph-2024-001215)
Supplement: online supplemental file 5 [file bmjph-3-1-s005.pdf]

GOVERNANCE COMMITTEE REGISTER

Maternity Homes Alliance

No.	HEALTH FACILITY NAME	GC MEMBER NAME	SEX	AGE	EDUCATION	NOTES	OCCUPATION	WOMAN OF REPRODUCTIVE AGE

GOVERNANCE COMMITTEE REGISTER

Maternity Homes Alliance

COMMUNITY WORKS INVOLVED IN								TYPE OF OFFICIAL	ZONE	CONTACT
SMAG/CBA	CHW	NUTRITION	PMTCT	NHC	CAREGIVER	MALARIA AGENT	TBA			
