# Supplementary material for: Implementation fidelity of a multisite maternity waiting homes programme in rural Zambia: application of the conceptual framework for implementation fidelity to a complex, hybrid-design study
Source: BMJ Public Health. 2025 Jan 16;3(1):e001215. doi: 10.1136/bmjph-2024-001215 (PMC11812881; doi:10.1136/bmjph-2024-001215)
Supplement: online supplemental file 6 [file bmjph-3-1-s006.pdf]

Supplemental File 6: Demographic characteristics of MWH users and the sub-set of experience survey respondents

	**MWH users** (N=3,206)	**Experience survey respondents*** (N=448)
Age (years), mean (SD)	24.4 (6.7)	24.1 (6.5)
Highest grade completed, mean (SD)	6.8 (2.7)	6.5 (2.9)
Marital status, n (%)		
Married/cohabiting	2747 (85.7)	376 (83.9)
Divorced/separated/widowed	89 (2.8)	16 (3.5)
Never married	355 (11.1)	41 (9.3)
Gravida, mean (SD)	3.3 (2.2)	3.1 (2.1)
Parity, mean (SD)	2.2 (2.1)	2.1 (2.1)
Distance of village to health facility, n (%)		
<5km	738 (23.0)	73 (16.3)
5-9km	938 (29.3)	146 (32.6)
≥10km	1,286 (40.1)	192 (42.9)
Village not indicated	244 (7.6)	26 (5.8)
Reasons for stay, n (%)		
Delivery	2,673 (83.4)	448 (100.0)
PNC	533 (16.6)	0 (0)
Length of stay (days), mean (SD)		
For delivery stays	13.0 (11.6)	21.3 (13.1)
For PNC stays	2.7 (4.6)	N/A
At time experience survey was administered	N/A	12.8 (9.3)

*11 experience survey respondents could not be linked to their demographic information.

SD = standard deviation; PNC = postnatal clinic
