# Supplementary material for: Implementation fidelity of a multisite maternity waiting homes programme in rural Zambia: application of the conceptual framework for implementation fidelity to a complex, hybrid-design study
Source: BMJ Public Health. 2025 Jan 16;3(1):e001215. doi: 10.1136/bmjph-2024-001215 (PMC11812881; doi:10.1136/bmjph-2024-001215)
Supplement: online supplemental file 7 [file bmjph-3-1-s007.pdf]

Supplemental File 7: Overall and facility level intervention duration & frequency

	Target	Overall	A	B	C	D	E	F	G	H	I	J
**Theoretical Framework Construct: Frequency**												
Total admissions	NA	3,206	314	232	211	328	465	586	366	283	122	299
Total nights stayed	NA	36,281	3,876	2,587	1,788	4,386	2,971	9,355	2,060	3,151	1,484	4,622
Occupancy rate	80%	52%	56%	37%	26%	66%	43%	134%	40%	47%	22%	69%
**Theoretical Framework Construct: Duration**												
Average length of stay (nights) for delivery stays	14.0	13.0	12.7	11.2	8.4	14.0	13.2	16.0	9.4	8.9	12.2	15.4
Average length of stay (nights) for PNC stays	NA	2.7	4.6	9.7	20.0	4.2	2.0	N/A	2.6	12.6	9.0	N/A

Note: Occupancy could be higher than 100% by repurposing the postnatal beds and using the companion mattresses
