# Supplementary material for: Implementation fidelity of a multisite maternity waiting homes programme in rural Zambia: application of the conceptual framework for implementation fidelity to a complex, hybrid-design study
Source: BMJ Public Health. 2025 Jan 16;3(1):e001215. doi: 10.1136/bmjph-2024-001215 (PMC11812881; doi:10.1136/bmjph-2024-001215)
Supplement: online supplemental file 8 [file bmjph-3-1-s008.pdf]

Supplemental File 8: Heat map depicting MWH users' perceptions and satisfaction with key elements of the Core MWH Model, including number of experience survey respondents per site and overall (Theoretical framework constructs: quality & participant responsiveness)

	**Overall** (n=448)	**A** (n=50)	**B** (n=39)	**C** (n=36)	**D** (n=54)	**E** (n=43)	**F** (n=73)	**G** (n=26)	**H** (n=48)	**I** (n=17)	**J** (n=62)
**Theoretical Framework Construct: Perceptions of MWH quality from experience survey respondents**											
**Overall perceived quality**	3.0	3.0	3.0	3.0	3.0	3.0	3.0	2.9	3.0	3.0	3.0
Perceived management and oversight of the MWH (scale)	3.0	3.0	3.0	3.0	3.0	3.0	3.0	3.0	2.9	3.0	3.0
Perceived cleanliness of the MWH (scale)	2.9	2.9	2.9	2.9	3.0	2.9	3.0	2.9	2.9	2.9	2.9
Perceived presence of staff (scale)	2.8	2.8	2.9	2.7	2.8	2.8	2.9	2.9	2.8	2.8	2.8
Perceived friendliness of staff (scale)	2.9	2.8	2.9	2.9	3.0	2.9	3.0	2.9	3.0	3.0	2.8
Perceived access to cooking area (scale)	2.5	2.4	2.3	2.4	2.3	2.9	2.2	2.8	2.9	2.8	2.8
Perceived crowdedness of MWH (scale)	2.7	2.8	2.6	3.0	2.9	2.9	2.2	3.0	3.0	3.0	2.7
Reportedly felt safe at MWH (scale)	3.0	3.0	3.0	2.9	3.0	2.8	3.0	3.0	3.0	2.9	3.0
Reported boredom while staying at MWH (scale)	2.8	2.7	2.8	2.8	2.8	2.8	2.8	2.6	2.7	2.7	2.8
Perceived cultural appropriateness of MWH (scale)	3.0	3.0	3.0	3.0	3.0	3.0	3.0	3.0	3.0	3.0	3.0
Reported participating in a class (%)	54.8	56.0	64.1	25.0	57.4	39.5	67.1	50.0	55.2	11.8	40.3
**Theoretical Framework Construct: Participant responsiveness from experience survey respondents**											
**General reported satisfaction with MWH stay**	2.9	2.9	2.9	2.9	2.9	2.9	3.0	3.0	2.9	2.8	2.9

Reported intent to recommend MWH to a friend (%)	99.2	98.0	100	97.2	98.1	100	100	100	100	100	100
Reported intent to return to MWH for future delivery (%)	96.0	94.0	100	94.4	96.3	95.3	100	88.5	97.9	94.1	95.2
Reported intent to return to MWH for PNC stay (%)	71.5	80.0	76.9	47.2	87.0	81.4	71.2	84.6	66.7	88.2	71.0

Scale Key: 3.00 2.99-2.70 2.69-2.40 2.39-2.10 2.09-1.80 <1.80

% Key: 100 85-99.9 70-84.9 55-69.9 40-54.9 <40

Note: Data were collected monthly from each implementation site from a purposive sample of MWH users who consented to participate in an experience survey after having stayed at least three nights at the MWH
