# Supplementary material for: Implementation fidelity of a multisite maternity waiting homes programme in rural Zambia: application of the conceptual framework for implementation fidelity to a complex, hybrid-design study
Source: BMJ Public Health. 2025 Jan 16;3(1):e001215. doi: 10.1136/bmjph-2024-001215 (PMC11812881; doi:10.1136/bmjph-2024-001215)
Supplement: online supplemental file 9 [file bmjph-3-1-s009.pdf]

Supplementary File 9. Strategies to facilitate implementation

	Overall	A	B	C	D	E	F	G	H	I	J
Total number of members	9.3	10	6	7	9	10	9	10	12	10	10
*Female membership, n (%)**	3.7 (39.8)	3 (30.0)	2 (33.3)	2 (28.6)	3 (33.3)	3 (30.0)	4 (44.4)	5 (50.0)	4 (33.3)	6 (60.0)	5 (50.0)
*Reproductive age women (15-49 years) membership, n (%)**	2.9 (31.2)	2 (20.0)	2 (33.3)	2 (28.6)	1 (11.1)	2 (20.0)	4 (44.4)	4 (40.0)	4 (33.3)	3 (30.0)	5 (50.0)
*Health facility staff membership, n (%)**	1.2 (12.9)	1 (10.0)	1 (16.7)	1 (14.3)	1 (11.1)	1 (10.0)	1 (11.1)	1 (10.0)	2 (16.7)	2 (20.0)	1 (10.0)
*Community-based volunteer membership, n (%)**	5.9 (63.4)	3 (30.0)	4 (66.7)	2 (28.6)	4 (44.4)	5 (50.0)	9 (100.0)	8 (80.0)	8 (66.7)	9 (90.0)	7 (70.0)
*Traditional leadership membership, n (%)**	0.8 (8.6)	1 (10.0)	1 (16.6)	0	0	1 (10.0)	1 (11.1)	0	1 (8.3)	2 (20.0)	1 (10.0)
Number of executive committee positions	4.8	5	5	4	5	6	4	6	4	4	5
*% female members on executive committee, n (%)***	2 (41.7)	2 (40.0)	1 (20.0)	2 (50.0)	1 (20.0)	2 (33.3)	1 (25.0)	4 (66.7)	2 (50.0)	0 (.)	5 (100.0)

* Denominators are total number of governance committee members at each implementation site and overall; ** Denominator is total number of executive committee members at each implementation site and overall.
